# A comprehensive bibliometric analysis (2000–2022) on the mapping of knowledge regarding immunotherapeutic treatments for advanced, recurrent, or metastatic cervical cancer

**DOI:** 10.3389/fphar.2024.1351363

**Published:** 2024-05-10

**Authors:** Yuanqiong Duan, Lin Yang, Wenxiang Wang, Peixuan Zhang, Kaiyu Fu, Wen Li, Rutie Yin

**Affiliations:** ^1^ Department of Obstetrics and Gynecology, West China Second University Hospital of Sichuan University, Chengdu, Sichuan, China; ^2^ Key Laboratory of Birth Defects and Related Diseases of Women and Children, Ministry of Education, Sichuan University, Chengdu, China

**Keywords:** cervical cancer, immunotherapy, bispecific antibodies, citespace, bibliometrics analysis

## Abstract

**Background:**

Despite extensive literature on therapeutic strategies for cervical cancer, a bibliometric analysis specifically focused on immunotherapy for advanced, recurrent, or metastatic (A/R/M) cervical malignancies remains unexplored. This study aims to address this gap by presenting a comprehensive overview that includes general characteristics, research focal points, the trajectory of evolution, and current emerging trends in this under-researched area.

**Methods:**

A systematic search was conducted using the Web of Science Core Collection (WOSCC) to identify articles related to A/R/M cervical cancer published between 2000 and 2022. Citespace and VOS viewer were the primary tools used to identify research focal points, intriguing future patterns, and to evaluate contributions and co-occurrences among authors, institutions, countries, and journals.

**Results:**

A total of 1,001 original articles were identified, involving 6,387 authors from 66 countries and 1,474 institutions, and published across 366 academic journals. The United States contributed most significantly. The most productive researcher was Van der Burg SH from Leiden University Medical Center. The International Journal of Cancer and Cancer Research were identified as the most productive and influential journals, respectively. Analysis of co-citation clusters highlighted 25 clusters, primarily focusing on potential predictive biomarkers, dendritic cell-based tumor vaccines, therapeutic HPV vaccinations, peptide-based cancer vaccines, tumor immune microenvironments, and adoptive cell transfer (ACT). The latest significant trends in A/R/M cervical cancer immunotherapy research included ACT, CAR-T, and immune checkpoint inhibitors (ICIs), as revealed by keyword and reference burst detection.

**Conclusion:**

This pioneering study provides a detailed landscape of immunotherapy research in A/R/M cervical cancer. It underscores the importance of global collaboration, enriches our understanding of the immunology of A/R/M cervical cancer, expands on potential beneficiaries of immunotherapy, and explores clinical applications of various therapies, including therapeutic vaccines, adoptive cell transfer, and ICIs, particularly in combination with established treatments such as chemotherapy, radiotherapy, and targeted therapy.

## 1 Introduction

Cervical cancer (CC) remains a significant global health challenge, ranking as the fourth most prevalent cancer among women worldwide. Despite the preventive potential of the human papillomavirus (HPV) vaccine and regular screening, GLOBOCAN 2020 reports about 604,000 new cases and approximately 341,000 deaths in 2020, emphasizing the urgent need for more effective treatment strategies (Ferrall et al., 2021; Sung et al., 2021). The prognosis for cervical cancer varies considerably with the stage at diagnosis: early-stage cancers, when detected and managed with surgery and chemoradiation, exhibit a 5-year survival rate exceeding 90%. In contrast, this rate drops to below 20% for patients with advanced or metastatic disease (Monk et al., 2022). Unfortunately, therapeutic advancements have not significantly improved survival rates since the 1970s (Feng et al., 2020; Siegel et al., 2022; Suran, 2022), underscoring the critical need for innovative therapeutic approaches, particularly for advanced, recurrent, or metastatic (A/R/M) cervical malignancies.

Immunotherapy is a rapidly evolving field that holds promise for treating a variety of malignancies, including cervical cancer. The unique interplay between persistent HPV infections and the immune system offers a valuable opportunity for targeted immunotherapy (Vinokurova et al., 2008; Pal and Kundu, 2019; Attademo et al., 2020). Additionally, factors such as high tumor mutation burden (TMB) and microsatellite instability (MSI) provide further rationale for employing immunotherapy in cervical cancer cases (Zhao et al., 2019; Tan et al., 2020). Early phase clinical trials have demonstrated the efficacy of this approach, showing sustained responses and a manageable safety profile (Duska et al., 2020; Frumovitz et al., 2020; Huang et al., 2022; Vicier et al., 2022; Xu et al., 2022). Moreover, numerous phase II and III studies are currently investigating the use of immunotherapy, both as a standalone treatment and in conjunction with chemotherapy and radiotherapy, for locally advanced and metastatic cervical cancer (Sugiyama et al., 2014; Harper et al., 2019; Mayadev et al., 2020; Takeuchi et al., 2020; O'Malley et al., 2021). Immunotherapy represents a significant anti-cancer strategy that harnesses and directs the host’s immune system to target cancer cells with increased specificity and efficacy, showing great potential to improve therapies and survival rates in advanced, recurrent, or metastatic cervical cancer.

The breadth of research on immunotherapy for A/R/M cervical cancer is extensive and continues to expand rapidly, posing challenges in distilling and synthesizing the vast amount of available information. In this context, bibliometric analysis proves to be an indispensable tool, offering a comprehensive perspective on the evolution of the field and facilitating the identification of pivotal trends and focal points within the research landscape (Chen, 2004). This methodology, which goes beyond traditional review mechanisms by utilizing mathematical and statistical techniques, provides a structured framework of knowledge, enabling more efficient assimilation of the literature (Ellegaard and Wallin, 2015; Chen and Song, 2019).

Despite the wealth of data, there is a noticeable dearth of bibliometric studies specifically targeting the immunology of A/R/M cervical cancer. To address this deficiency, our study undertakes a thorough bibliometric analysis of the literature from 2000 to 2022. We aim to deliver a detailed overview of the existing knowledge base and the emergent trends in the immunotherapy of A/R/M cervical carcinoma. This analysis will yield both quantitative and qualitative insights, laying the foundation for future research in this vital field.

## 2 Materials and methods

### 2.1 Origins of bibliometric data

Given the broad scope of the Science Citation Index Expanded (SCI-E) and its relevance to our study, we selected it as the primary database for our bibliometric analysis (Lawson McLean, 2019; Yao et al., 2020). We conducted a systematic literature review of publications from 2000 to 2022. To ensure the precision and relevance of our dataset, we utilized two principal search terms: ‘A/R/M cervical cancer’ (Strategy A) and ‘immunotherapy’ (Strategy B). A Boolean search algorithm using “A AND B" (Strategy C) refined our search, ensuring that all articles retrieved were directly relevant to immunotherapy for cervical cancer.

**FIGURE 1:**
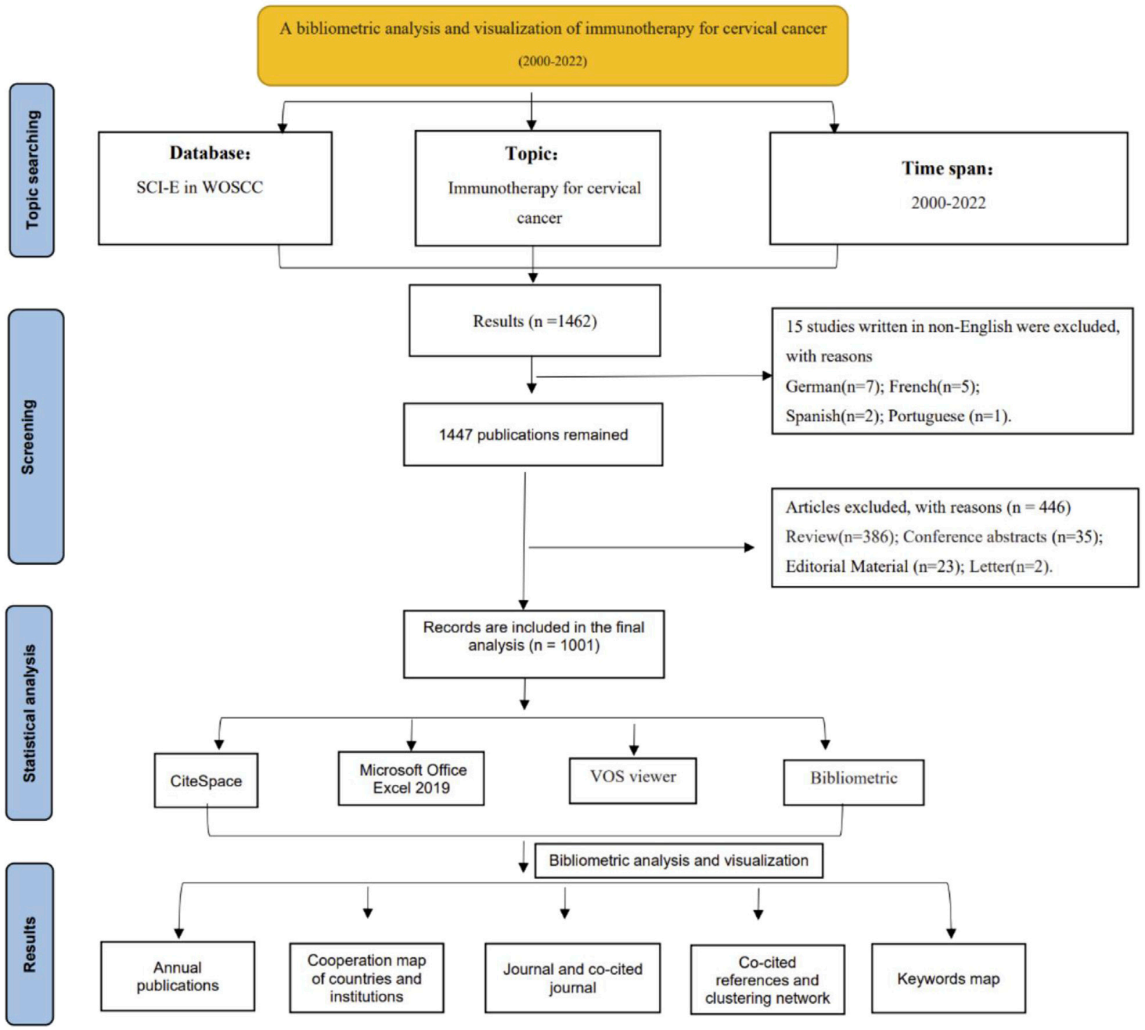
Flowchart of the exclusion and incorporation of literature.

The literature retrieval process was rigorously conducted by two researchers independently. In cases of disagreement, issues were resolved through discussions with the corresponding author, ensuring a balanced and comprehensive selection of articles. Additionally, we restricted our search to articles published in English to maintain clarity and uniformity in our analysis, thereby enhancing the academic rigor of our study. This decision was also supported by the tendency of journal articles to introduce new and innovative findings. Furthermore, our study was meticulously structured according to PRISMA guidelines to ensure a robust and transparent research methodology. The procedural flow of our study, from the initial data collection to the final article selection, is illustrated in the flowchart presented in Figure 1.

### 2.2 Methods of analysis and visualization

Bibliometric analysis, an emerging field, employs visualization and statistical methods to examine structures and trends within specific subjects or domains (Jiang et al., 2018). Its primary objective is to identify significant nodes and extract valuable insights from extensive data repositories. For data processing and visualization, we primarily utilized CiteSpace and VOS Viewer for their distinctive features and advantages, alongside other industry-standard tools such as NetDraw, HistCite, and SCI2 (Qin et al., 2020).

CiteSpace, a publicly accessible Java application, is specifically designed to uncover patterns and trends in scientific literature. Developed by Professor Chaomei Chen, it aids in identifying critical junctures and turning points in the evolution of disciplines, enabling the visualization of knowledge domains (Synnestvedt et al., 2005). CiteSpace excels at illustrating collaboration networks, identifying key elements, mapping internal structures, forecasting trends, and tracking changes within specific fields (Chen, 2004). We used CiteSpace to analyze and depict the co-presence of nations, regions, and organizations, as well as trends in frequently occurring keywords, co-cited references, and bursts in reference citations.

The VOS Viewer, developed by the Center for Science and Technology Studies at Leiden University, facilitates the creation, display, and exploration of maps based on network data (van Eck and Waltman, 2010). This software streamlines the visualization of scientific networks. Employing VOS Viewer (version 1.6.18.0), we effectively identified productive academic journals, their co-citations, and associated knowledge maps through bibliographic data analysis. Additionally, Microsoft Office Excel 2019 was employed for annual publication analysis and database management. The impact factor (IF), H-index, and Journal Citation Reports (JCR) rankings of journals for 2022 were obtained from the Incites Journal Citation Reports of the Web of Science and the bibliometric online analysis platform (https://bibliometric.com/app).

## 3 Results

### 3.1 Annual publication trends

**FIGURE 2:**
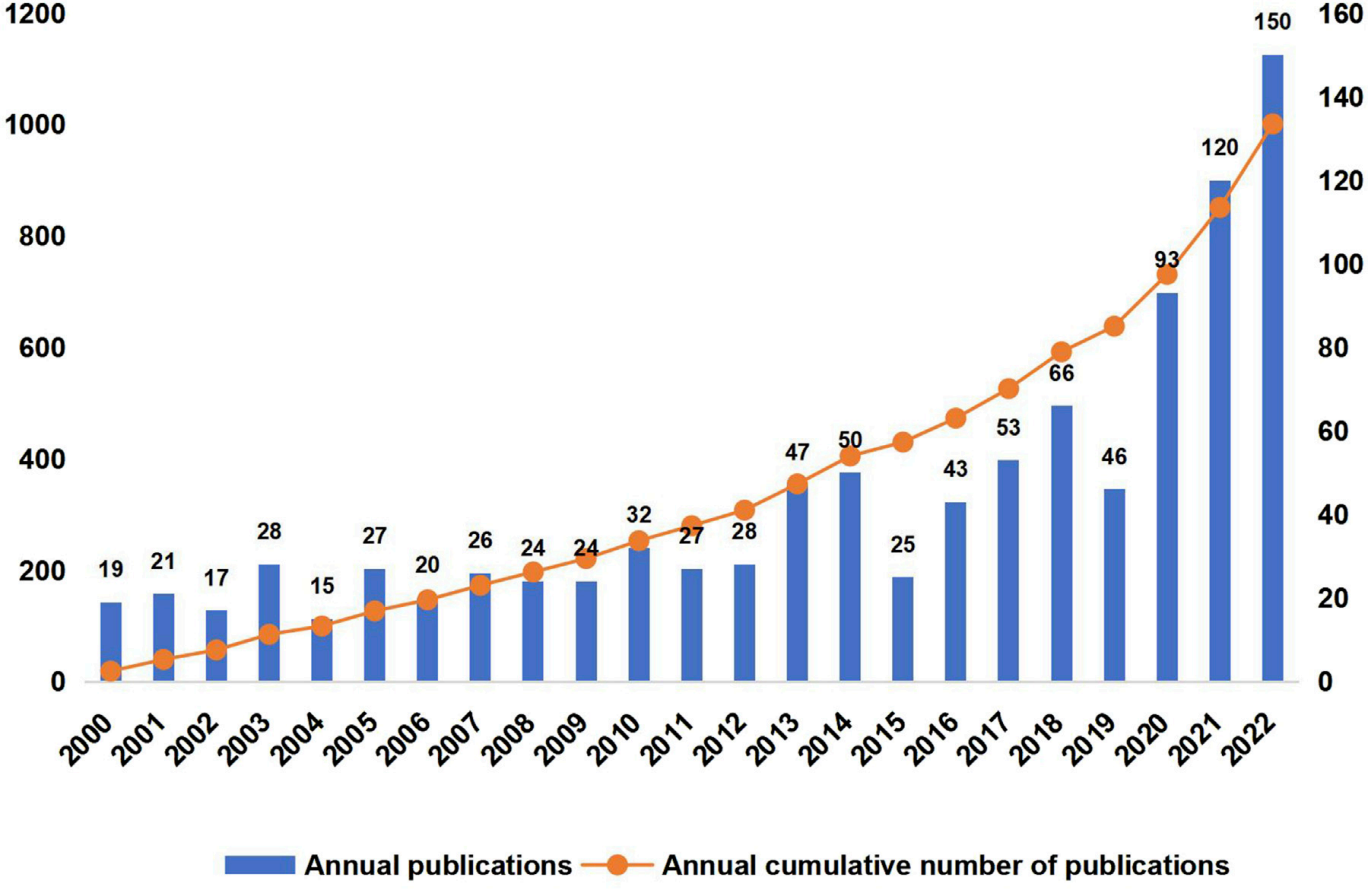
Trends in annual publications concerning immunotherapeutic interventions for advanced, recurrent, or metastatic (A/R/M) cervical cancer.

From 2000 to 2022, we identified 1,001 distinct articles through our data collection strategy, representing a substantial corpus of research in the field of A/R/M cervical cancer immunotherapy. Figure 2 illustrates the annual distribution of these articles. The initial phase, spanning 2000 to 2019, demonstrates a steady increase in publication volume. The subsequent phase, from 2020 to 2022, is characterized by a significant surge in research activity, reflecting a heightened focus on this area.

### 3.2 Map of international and institutional cooperation

#### 3.2.1 Contributions of countries

**FIGURE 3:**
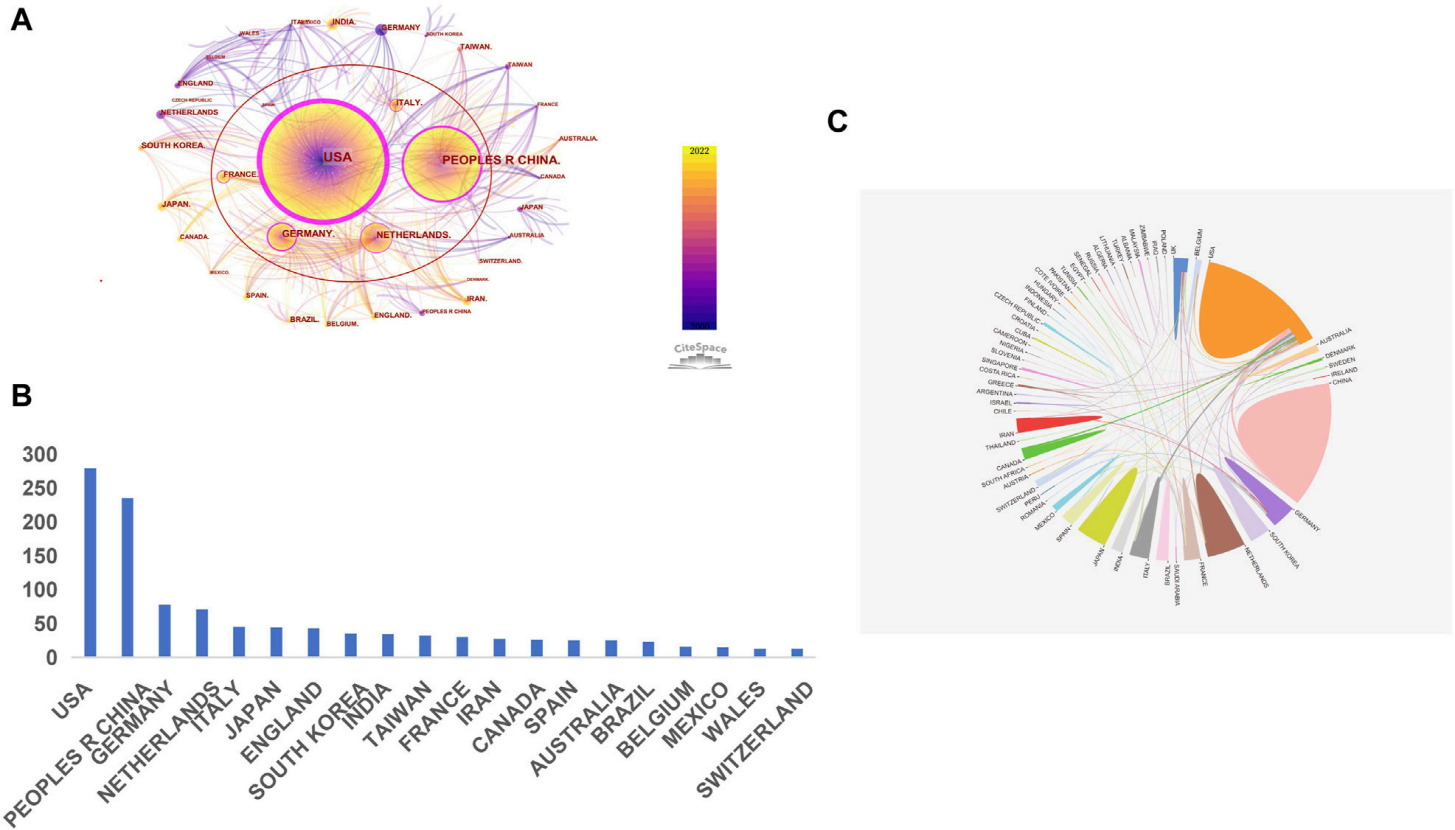
A map of international interactions based on a geographic analysis of collaboration **(A)**. Visualization of the network of country co-occurrences (There are 66 nodes and 726 connecting connections in the network. The magnitude of the nodes and the length of the lines represent, respectively, the quantity of a country’s publications and the extent of its cooperation within a given geography. Variable-colored lines represent various years, and the presence of a red ellipse indicates an increase in citations or frequency for a particular country.) **(B)**. Count of publications for the top 20 countries **(C)**. Interactions and collaborations among countries.

The global endeavor in cervical cancer immunotherapy involves contributions from sixty-six countries. Utilizing CiteSpace, we visualized the collaboration network, displayed in Figure 3A, which highlights the extensive contributions from various nations. In this network, the size of each node corresponds to the total number of publications from specific countries, institutions, or authors (Chen et al., 2012), with connecting lines indicating citation relationships. Notably, the United States, China, Germany, the Netherlands, France, Italy, and Spain are marked in purple, signifying their central role and potential to drive research breakthroughs. Figure 3B shows that the United States leads with 279 publications, accounting for 27.9% of the total, followed by China with 235 publications (23.5%). Other prominent contributors include Japan, Germany, the Netherlands, and Italy. Figure 3C depicts the patterns of international collaboration, with the United States and China, as well as the United States and Italy, being the most frequent collaborators.

#### 3.2.2 Contributions of institutions

**FIGURE 4:**
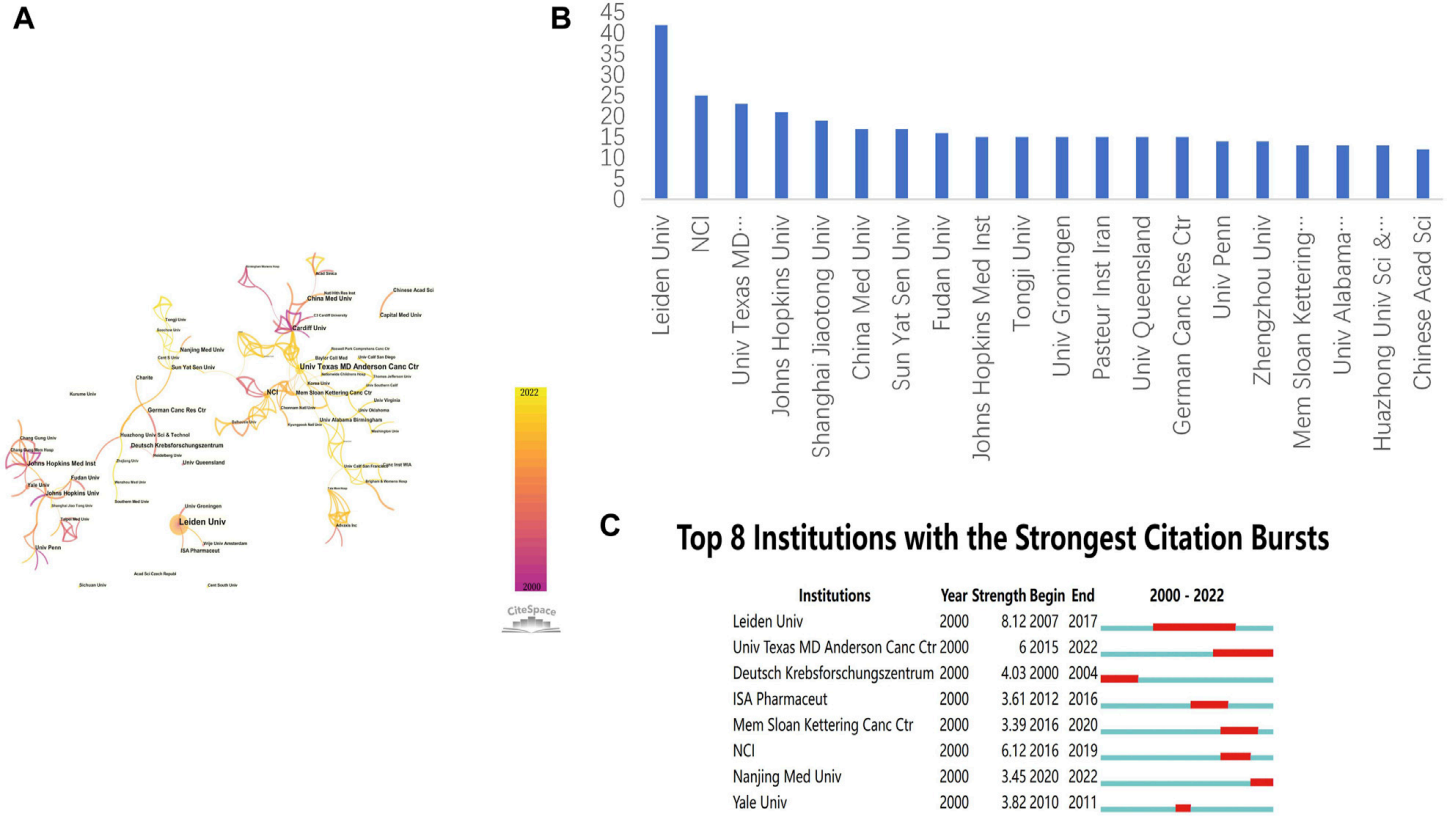
Collaborative Infrastructure Map of Institutions **(A)**. Co-occurrence network among institutions (the network consists of 1,474 nodes and 2,940 connecting lines. The sizes of the nodes and lines correspond to the quantity of an institute’s publications and the scale of cooperative interactions within the institute, respectively. Lines of assorted colors correlate to different years.) **(B)**. Publication quantity for the top 20 institutions **(C)**. Top 8 institutions experiencing the most pronounced citation surges (Red bars denote periods when a certain institution garners frequent citations, sorted by the duration of the burst.).

The field of immunotherapy for advanced, recurrent, or metastatic (A/R/M) cervical cancer has attracted contributions from 1,474 academic institutions globally. Figure 4A, generated using CiteSpace, displays a network map of these institutions, illustrating their distribution and interconnections. Leiden University is highlighted as the foremost contributor with 42 publications, as depicted in Figure 4B. Other prominent institutions include the National Cancer Institute (NCI), the University of Texas MD Anderson Cancer Center, Johns Hopkins University, and Shanghai Jiao Tong University, with the majority of the top ten institutions located in the US and China. This distribution underscores the pivotal role these countries play in advancing research in immunotherapy for A/R/M cervical cancer. Figure 4C lists the eight most cited institutions, ranked by the duration of their citation bursts, further highlighting the significant impact of these organizations.

### 3.3 Journals and co-cited journals

Utilizing VOS Viewer for the analysis of co-citations and identification of key journals in A/R/M cervical cancer immunotherapy, we observed significant publication and citation trends. As indicated in Supplementary Table S1, the International Journal of Cancer leads with 3.1% of the total publications (31 publications), followed by Cancer Immunology Immunotherapy, Vaccine, Gynecologic Oncology, and Oncoimmunology, each making substantial contributions to the field. Notably, four of the top ten journals boast impact factors above five and H-indexes exceeding one hundred, highlighting their significant influence within the scientific community. These journals include Clinical Cancer Research (IF: 11.5; H-Index: 292), Cancer Research (IF: 13.2; H-Index: 411), International Journal of Cancer (IF: 6.4; H-Index: 212), and Cancer Immunology Immunotherapy (IF: 5.8; H-Index: 104). Furthermore, Cancer Research leads in citation volume with 630 citations, as detailed in Supplementary Table S2, followed by Clinical Cancer Research and the International Journal of Cancer.

### 3.4 Authors and co-cited authors

**FIGURE 5:**
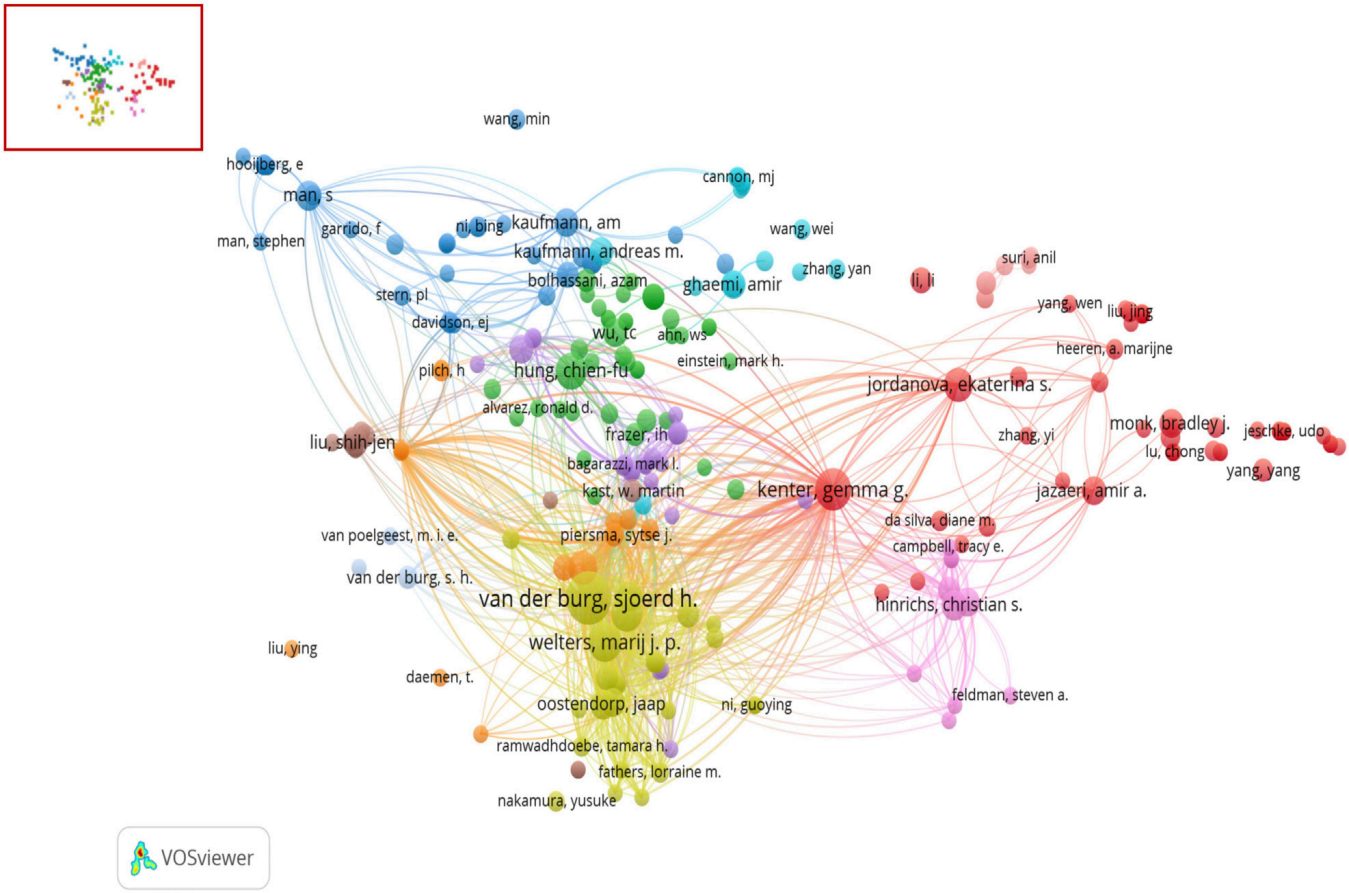
Collaborative network map among authors.

A total of 6,387 authors have contributed to the field of immunotherapy for A/R/M cervical cancer. The ten most productive researchers are listed in Supplementary Table S3, with Van der Burg SH from Leiden University Medical Center leading with 25 publications. This author also ranks highest in total citations, amassing 1,878 citations, followed closely by Kenter GG and Melief CJM. Notably, Melief CJM has the highest H-index, underscoring the impact and relevance of his contributions. This comprehensive analysis underscores the critical roles played by authors such as Van der Burg SH and Melief CJM in advancing this field. Figure 5 depicts the emergence of dominant academic clusters and the patterns of collaboration within and between these groups. For example, Van der Burg SH (yellow cluster) is shown to collaborate closely with Welters MJP, and cross-cluster collaborations are evident between Van der Burg SH and other key researchers such as Kenter GG (red cluster) and Hung CF (green cluster). These collaborative networks illustrate the interconnected nature of research in this field and emphasize the importance of academic teamwork in advancing knowledge.

### 3.5 Co-cited references and networking cluster analysis

Co-cited references, which are scholarly works frequently cited together, establish a knowledge base that reflects the research community’s focal points and trends (Synnestvedt et al., 2005; Lu et al., 2020). To examine this aspect within the realm of A/R/M cervical cancer immunotherapy, we performed a co-citation analysis on 1,001 articles using CiteSpace. Supplementary Table S4 displays the ten most-cited references in this domain. Notably, “Global cancer statistics 2018: GLOBOCAN estimates of incidence and mortality worldwide for 36 cancers in 185 countries” stands out as the most cited, offering essential insights into the global impact of cervical cancer. Other significant co-cited references include studies on the “safety and efficacy of PD-1 inhibitors,” “integrated genomic and molecular characterization of cervical cancer,” and “therapeutic vaccines,” each playing a crucial role in advancing immunotherapy for this condition.

**FIGURE 6:**
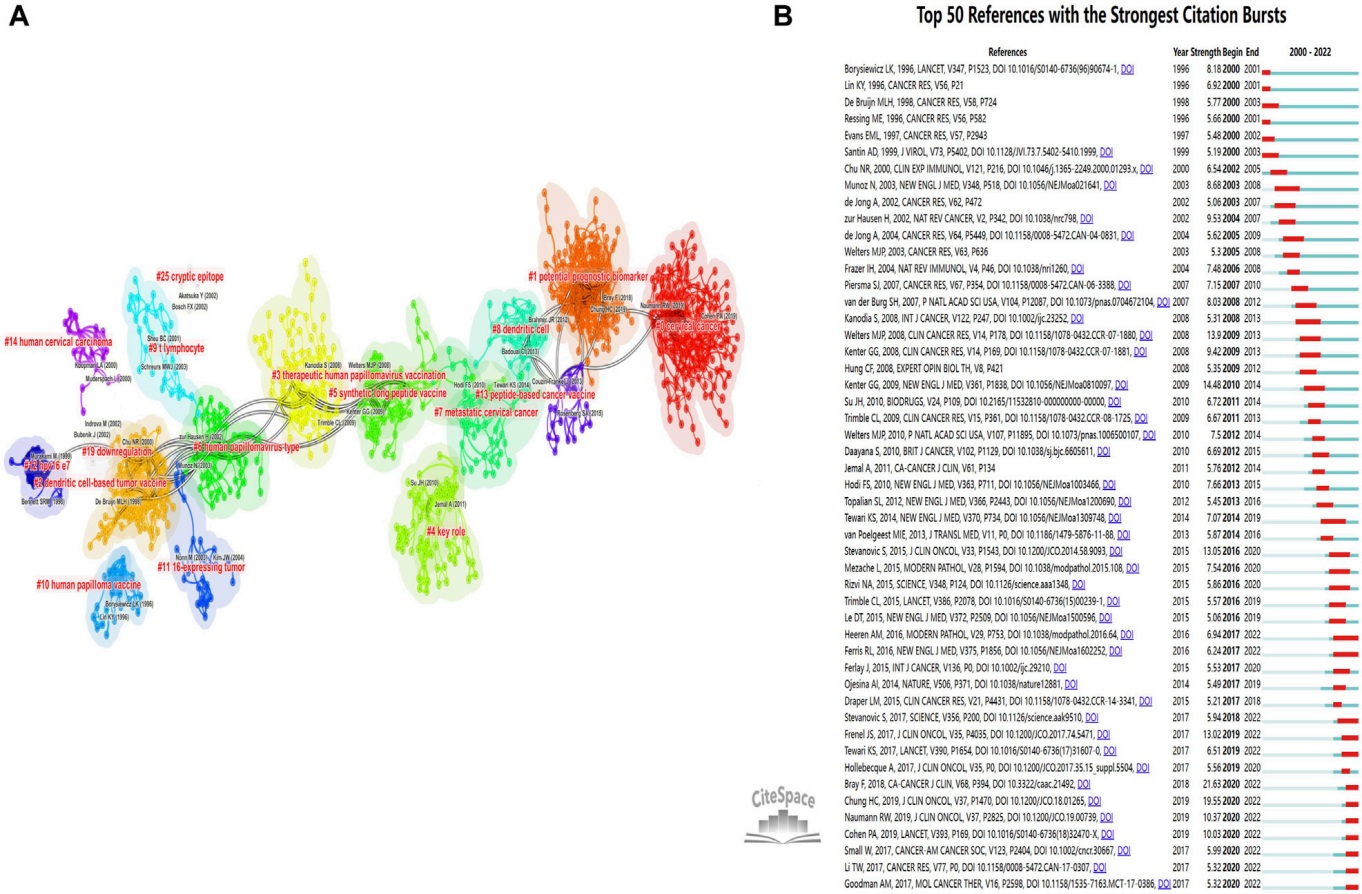
Visual representation of co-cited references in the domain of A/R/M cervical cancer immunotherapy **(A)**. Visualization of clusters formed by co-cited references **(B)**. The top 50 most frequently cited references (Red bars are sorted based on the starting year of the citation surge, indicating strong citation surges.).

We employed CiteSpace to generate a co-citation map, illustrating the interconnections among co-cited references and clustering them to delineate research boundaries in A/R/M cervical cancer immunotherapy. This analysis produced 25 distinct clusters, visualized in Figure 6A. The clustering structure, characterized by a mean silhouette value of 0.9178 and a modularity Q score of 0.8162, demonstrates robust and meaningful groupings. Each cluster represents a specific research area within the field. The largest identified clusters were “cervical cancer” (cluster #0), “potential prognostic biomarker” (cluster #1), “dendritic cell-based tumor vaccine” (cluster #2), and “therapeutic human papillomavirus vaccination” (cluster #3). Other noteworthy clusters include “peptide-based cancer vaccine,” “T-lymphocyte,” “MHC class-I downregulation,” “synthetic long peptide vaccine,” and “HPV 16 E7,” which suggest significant advancements or breakthroughs in this domain. Furthermore, we analyzed the citation dynamics of these references, highlighting the top 50 with notable citation surges as depicted in Figure 6B. These surges underscore references that are gaining traction over time within the research community. Notably, the reference by Bray F. (2018) exhibited the highest citation strength at 21.63, followed by Chung et al. (2019) at 19.55 and Kenter et al. (2009) at 14.48, underscoring their substantial impact in the field.

### 3.6 Key topics of research hotspots

In bibliometrics, keywords serve as indicators of trends and focal points within a research domain (Li et al., 2016). They capture the essence of an academic article and, when analyzed collectively, reveal the central themes and directions of a field. Networks of co-keyword and keyword co-occurrence commonly feature overlapping keywords across articles on related topics, allowing for an in-depth analysis of the field’s topological properties and their evolution.

#### 3.6.1 Analysis of clusters and co-occurrence of keywords

**FIGURE 7:**
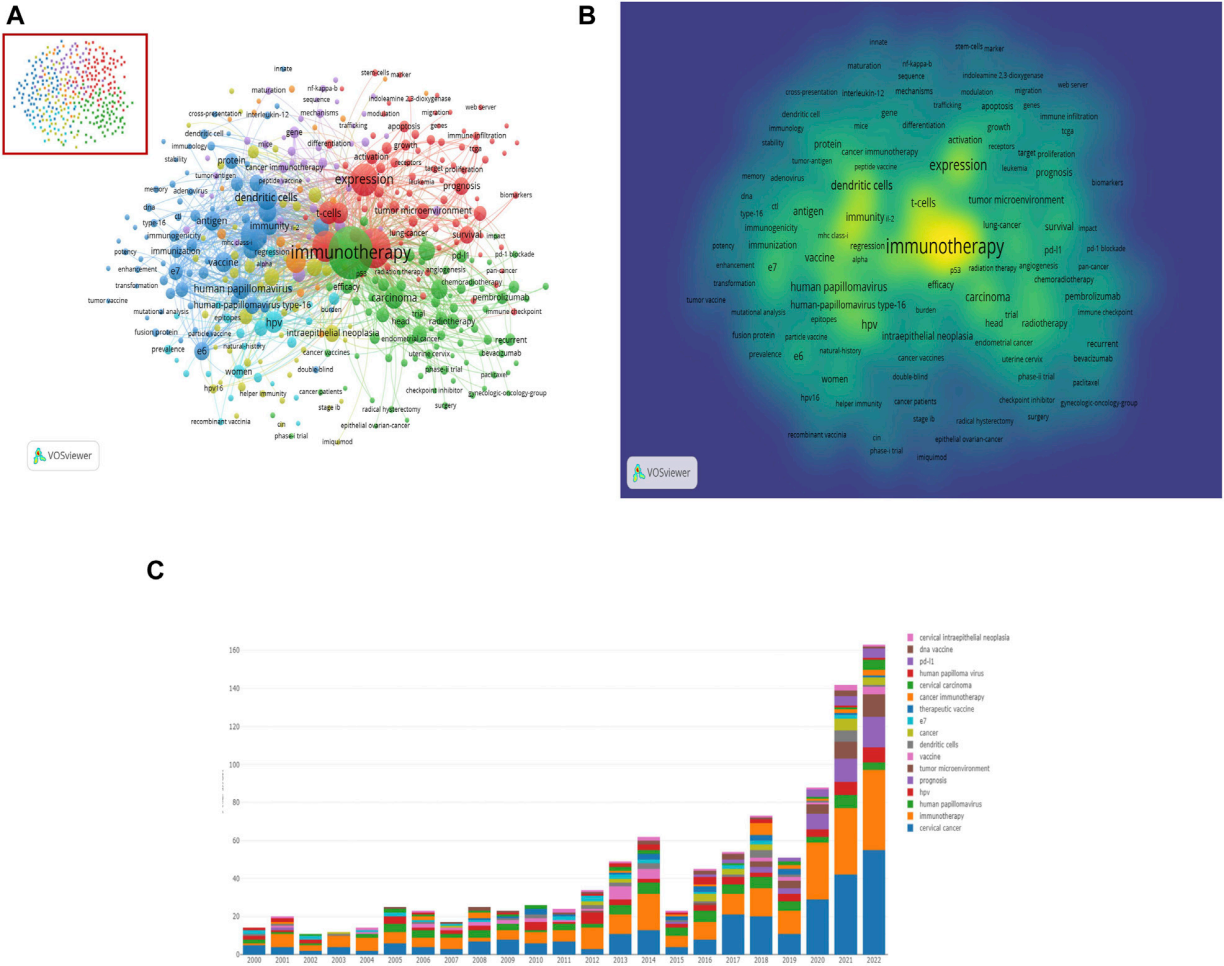
Keyword map in the domain of A/R/M cervical cancer immunotherapy **(A)**. Co-occurrence network and clusters of keywords (There are 641 keywords with a relevance score of at least 0.081. These keywords form a co-occurrence network and are organized into 7 clusters, resulting in a total of 42107 links. The connections and sizes of the nodes and words reflect the co-occurrence frequencies, indicating the co-occurrence relationship. Nodes of the same color serve as cluster representations) **(B)**. The keyword density map (Correlations exist between the co-cited frequency and the dimensions of the word, the roundness of the size, and the opacity of the yellow. It is capable of locating high-frequency co-occurrence terms, which indicate research centers.) **(C)**. Annual high-frequency keywords.

To visualize keyword co-occurrence maps in the field of A/R/M cervical cancer immunotherapy, we utilized VOS Viewer (van Eck and Waltman, 2010). This tool enabled the creation of a keyword density map and a network illustrating keyword co-occurrences (Figure 7A, B). Additionally, we employed an online bibliometric analysis platform to identify and categorize annual high-frequency keywords (Figure 7C). The cluster analysis of these keywords provides insights into the knowledge structure of this field.

As depicted in Figure 7A, the network was divided into seven clusters based on the strength of the connections between co-occurring terms. Cluster 1 (green) contains 86 keywords focused on immune checkpoint inhibitors and related mechanisms. These keywords include activation, apoptosis, checkpoint blockade, B7-H1 (PD-L1), CTLA-4, FoxP3, tumor immune microenvironment, neoantigens, and IDO. Cluster 2 (red), with 82 keywords, primarily discusses immunotherapy strategies such as adoptive cell transfer (ACT), antitumor activity, immune checkpoint inhibitors, cytolytic T-lymphocytes, tumor-infiltrating lymphocytes, and CAR-T immunotherapy. Cluster 3 (blue) consists of 74 terms related to HPV vaccines and includes adenovirus, E6, E7, antigen presentation, antitumor immunity, cancer vaccine, DNA vaccine, and therapeutic vaccine. Cluster 4 (yellow) features 51 terms mostly associated with vaccination strategies, including adoptive transfer, antigens, cytokines, CD4^+^, cytotoxic T-lymphocytes, IL-12, peptides, and helper immunity. Cluster 5 (purple) focuses on 47 terms concerning adoptive immunotherapy, such as biomarkers, monoclonal antibodies, natural killer cells, toll-like receptors, peptide vaccines, MHC class-I downregulation, and CD8^+^ T cells. Cluster 6 (light blue) centers on HPV infection, comprising 23 terms like CD4, CD8, cell responses, CIN, regression, and natural history. Finally, Cluster 7 (orange) contains 19 terms related to immunoregulation, including regulatory T-cells, TGF-beta, hypoxia, and more.

#### 3.6.2 Burst detection and overlay visualization of keywords

**FIGURE 8:**
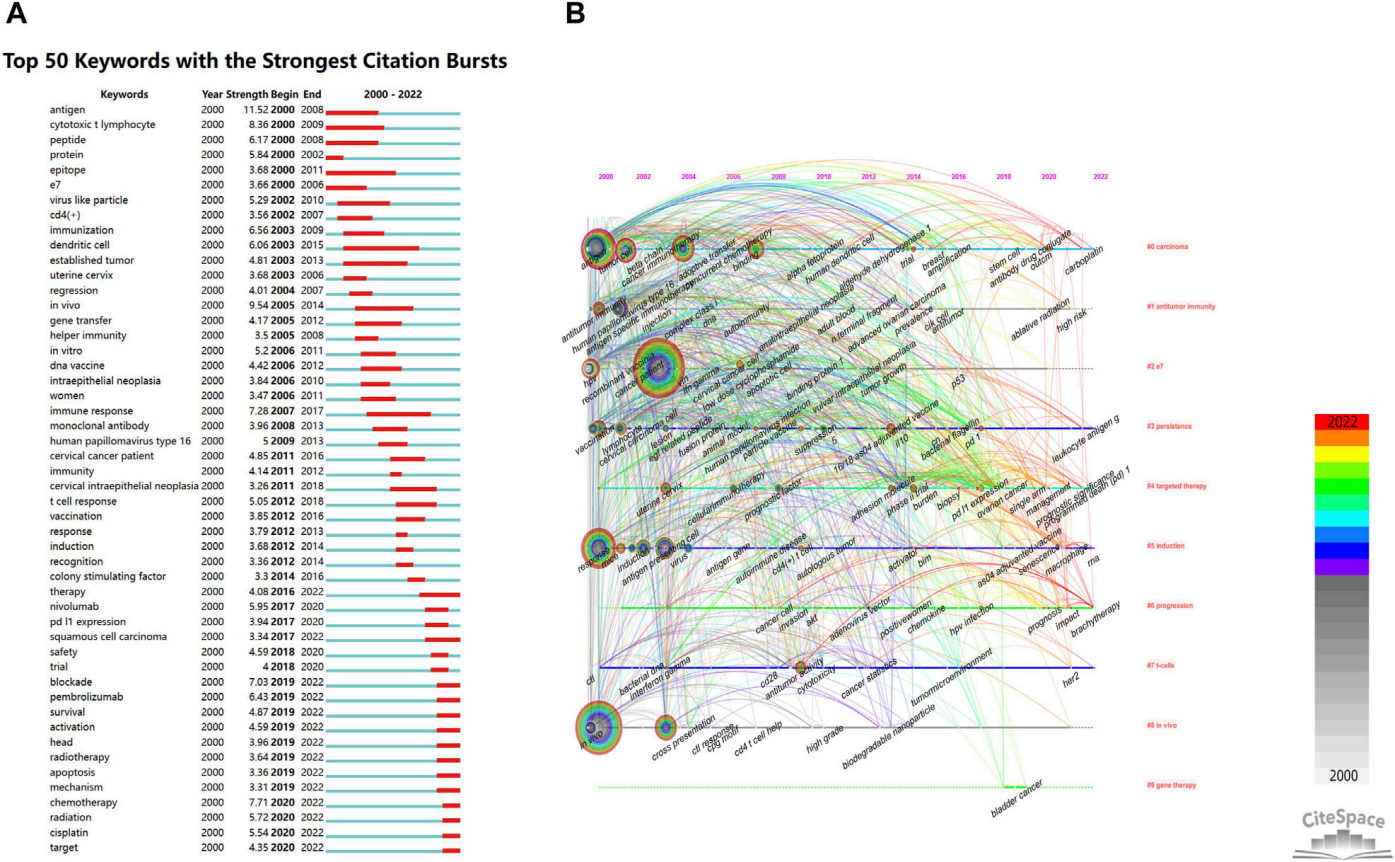
Scholastic cartography of keyword bursts and overlay visualization in the domain of immunotherapy for A/R/M cervical cancer **(A)**. Top 50 keywords with the strongest citation bursts (Red bars arranged according to the initial year of explosion indicate which keywords are frequently cited during a specific time period.) **(B)**. The temporal perspective of co-citation clusters (A time map represents the co-citation network for the given keyword from 2000–2022. A horizontal line represents the year of publication of each node in each cluster of this time map. Identifying the most recent and actively cited clusters is feasible within the time frame of 2019–2022).

CiteSpace’s keyword burst detection function is instrumental in identifying frequently appearing keywords within specific time frames, highlighting emerging topics in the field. As illustrated in Figure 8A, significant research hotspots over the past decade include vaccination (strength, 3.85; 2012–2016), PD-L1 expression (strength, 3.94; 2017–2020), nivolumab (strength, 5.95; 2017–2020), blockade (strength, 7.03; 2019–2022), pembrolizumab (strength, 6.43; 2019–2022), and apoptosis (strength, 3.36; 2019–2022). These terms indicate ongoing areas of research emphasis. Furthermore, an overlay visualization of the co-citation network’s keyword timeline (2000–2022) was generated using CiteSpace, providing an in-depth view of the evolution of these keywords over time, as depicted in Figure 8B.

## 4 Discussion

### 4.1 General information

This bibliometric analysis offers a pioneering exploration of the intellectual foundation and research fronts of immunotherapy for A/R/M cervical cancer. Employing bibliometric and visual analysis techniques, this study examines global research trends from 2000 to 2022, encompassing publication patterns, contributing countries, institutions, journals, and key terms. The search captured 1,001 Web of Science publications involving 6,387 authors from 66 countries and 1,474 institutions, published across 366 scholarly journals. The increasing recognition of this field is largely attributed to significant innovations, such as the “KEYNOTE-158″Phase II study, which assessed the safety and efficacy of Pembrolizumab in previously treated advanced cervical carcinoma (Chung et al., 2019). A notable surge in publications over the past 3 years underscores the field’s growing relevance and its potential for future advancements.

Only two of the 66 countries involved have published over 100 papers, with the United States and China clearly leading in A/R/M cervical cancer immunotherapy research. The United States, with a centrality score of 1.15, has played a pivotal role in fostering international collaboration. China stands out as the only developing country among the top five most productive nations. Among research institutions, Leiden University in the US is notable for having the highest number of publications and attracting significant attention. Economic disparities and varying national policies likely contribute to regional differences in the production of knowledge on cervical cancer immunotherapy. As illustrated in Figure 3A and 4A, while extensive collaboration is evident among many nations and organizations, others remain less connected. Therefore, it is recommended that countries and institutions involved in similar research increase their collaborative efforts to further develop and enhance this field.

### 4.2 Knowledge base

The utilization of co-citation analysis has been instrumental in delineating a shared body of knowledge among highly cited sources, underscoring the interconnectedness of seminal works within the field (Chen, 2004; Chen and Wu, 2017). In this bibliometric study, the co-citation cluster network and the ten most-cited references are displayed in Figure 6A and Supplementary Table S1, respectively. Notably, there is a significant overlap between our top 10 co-cited sources and the top 50 sources recognized for frequent citation bursts. Moving forward, we plan to conduct a comprehensive review of the key literature on cervical cancer immunology, focusing on the top ten sources that are frequently cited together. This analysis will aim to provide a holistic view of the subject, encompassing both fundamental scientific research and clinical studies.

Currently, preventive vaccines provide almost complete protection against persistent HPV infections and the development of severe genital lesions (Lowy and Schiller, 2006; Wheeler, 2007). However, women already infected with HPV strains covered by the vaccine do not benefit from it (Hildesheim et al., 2016). Consequently, there is a need for a therapeutic vaccine that can protect infected individuals. T-cell-based immunotherapeutic strategies effectively target the HPV16 E6 and E7 oncoproteins, which are present in all cervical cancer cells caused by HPV16(Hung et al., 2007; Vici et al., 2016; Saxena et al., 2021). *Clinical Cancer Research* published the ninth most-cited study in 2008 (Welters et al., 2008), which discussed a synthetic long-peptide vaccine targeting HPV16 E6 and E7. This vaccine significantly increases CD4^+^ and CD8^+^ T-cell responses specific to HPV16, targeting various epitopes. Following this, *the New England Journal of Medicine* published the eighth co-cited study (Kenter et al., 2009), which demonstrated that women with grade 3 vulvar intraepithelial neoplasia caused by HPV-16 could achieve clinical responses after being vaccinated with a similar synthetic long-peptide vaccine. This innovation has been pivotal in advancing the development of therapeutic vaccines based on oncoproteins for cervical cancer. Over recent decades, there has been a resurgence in the development of therapeutic cancer vaccines. Enhanced understanding of tumor-associated antigens, advances in innovative antigen delivery methods, and insights into the natural immune response have collectively improved vaccine design (Sahin and Türeci, 2018; Saxena et al., 2021). Nevertheless, tumor-induced immunosuppression and the emergence of resistance to immune responses pose significant challenges in achieving effective treatments. Current efforts to target HPV E6 and E7 with therapeutic vaccines have yet to yield significant success in treating A/R/M cervical cancer (van Elsas et al., 2020; Saxena et al., 2021). To achieve a substantial clinical impact, it will be necessary to design a multi-therapeutic approach tailored to the specific challenges of A/R/M cervical cancer. Integrating vaccines with other treatments, such as chemotherapy, radiation therapy, or additional immunotherapies, may enhance treatment outcomes.

Adoptive T cell therapies (ACT), also known as T-cell-based vaccines, entail the extraction of T cells from the host, followed by their enhancement and reinfusion. This process allows T cells to selectively proliferate and attack tumor antigens *ex vivo*, ultimately inducing tumor regression (Rohaan et al., 2019; Sukari et al., 2019). ACT primarily utilizes T cells modified with chimeric antigen receptors (CARs), engineered T-cell receptors (TCRs), and tumor-infiltrating lymphocytes (TILs) (Draper et al., 2015). A study published in *the Journal of Clinical Oncology* highlighted ACT as a novel treatment for metastatic cervical cancer (Stevanović et al., 2015). In a pivotal study, three of nine patients receiving a single infusion of selected TILs exhibited objective tumor responses—two complete and one partial. The complete responses were sustained for 22 and 15 months, respectively. Importantly, TILs targeting the HPV oncogenes E6 and E7 have proven effective in reducing tumor sizes in advanced cervical cancer cases. Alternatively, ACT can use genetically engineered TCRs designed to target specific tumor antigens (Draper et al., 2015). A completed Phase I/II trial suggested that E6 TCR therapy could induce remission in HPV-associated epithelial malignancies. Consequently, further research into this treatment is imperative (Klebanoff et al., 2016; Doran et al., 2019). Several ongoing clinical trials are evaluating the efficacy of HPV oncoprotein-specific TCR T cells in treating cervical cancer, with results expected soon (NCT03578406, NCT04476251, and NCT02858310). ACT represents a highly personalized, innovative therapy that may overcome the limitations of traditional chemotherapy in treating A/R/M cervical cancer.

Immunotherapeutic strategies targeting immunological checkpoint molecules, such as CTLA-4 and PD-1 on activated T cells, have induced complete and durable responses in various cancers, including melanoma and bladder malignancies (Wheeler, 2007; Powles et al., 2014). A study in *Modern Pathology* found that squamous cell carcinoma and adenocarcinoma differentially express PD-L1, with squamous cell carcinomas showing significantly higher PD-L1 expression and tumor-associated macrophage levels than adenocarcinomas. These findings underscore PD-L1’s critical role in cervical cancer’s immune evasion and support therapeutic targeting of the PD-1/PD-L1 pathway (Heeren et al., 2016). Between 2017 and 2019, the *Journal of Clinical Oncology* published several trials confirming these observations (Frenel et al., 2017; Chung et al., 2019; Naumann et al., 2019). Notably, pembrolizumab monotherapy has demonstrated sustained anticancer efficacy and tolerability in patients with advanced cervical cancer (Chung et al., 2019). Consequently, the FDA expedited approval of pembrolizumab for patients with advanced, PD-L1-positive cervical cancer showing progression during or after treatment. In 2017, *Nature* reported a record number of genomic studies on cervical cancer, revealing critical mutations in genes such as CASP8, SHKBP1, ERBB3, HLA-A, and TGFBR2. The studies also highlighted APOBEC mutation patterns, the impact of BCAR4 lncRNA on lapatinib response, and new amplifications in immune targets CD274/PD-L1 and PDCD1LG2/PD-L2. These insights, derived from a comprehensive molecular analysis of 228 primary cervical cancers, suggest potential new therapeutic targets and advocate for personalized treatment strategies for advanced/recurrent/metastatic cervical cancers. In conclusion, the advancement of immunotherapeutic approaches targeting the PD-1/PD-L1 pathway represents a significant breakthrough in improving cervical cancer treatments. The integration of these innovations with personalized therapy and continued research efforts is redefining the possibilities of cancer treatment, offering a promising future for individuals facing this challenging disease.

### 4.3 Progression of hotspots and emerging subjects

The recent advances in therapeutic development, particularly in harnessing the host immune system to manage and potentially eliminate cancer, are reflected in the prevalent keywords in current research. Several studies (Cohen et al., 2019; van Elsas et al., 2020; Wang et al., 2020; Ferrall et al., 2021; Norberg and Hinrichs, 2023) demonstrate a growing interest within the scientific community in integrating immunotherapy with traditional cancer treatments such as radiotherapy and chemotherapy. The goal of combining these modalities is to improve therapeutic outcomes and develop more effective cancer control strategies. To highlight these advancements, we present key summaries and discussions from the 2023 ASCO conference, focusing on significant breakthroughs in the field of immunotherapy for A/R/M cervical cancer.

The KEYNOTE-826 study (NCT03635567), highlighted by Bradley J. Monk at the 2023 ASCO conference, reported compelling survival results (Monk et al., 2023). Pembrolizumab achieved a final overall survival of 28.6 months in patients with PD-L1 CPS≥1, 26.4 months across all participants, and a peak of 29.6 months for those with PD-L1 CPS≥10. Progression-free survival was noteworthy, reaching 10.5 and 10.4 months for PD-L1 CPS≥1 and all patients, respectively. These outcomes were statistically significant, regardless of bevacizumab co-administration. In patients with PD-L1 CPS≥1, pembrolizumab significantly increased the objective response rate (68.5% *versus* 50.9% for placebo) and complete response rate (25.6% *versus* 14.5% for placebo). These results affirm the combined use of pembrolizumab and chemotherapy, with or without bevacizumab, as a first-line treatment for advanced/recurrent/metastatic cervical cancer, echoing previous interim findings (Colombo et al., 2021). Furthermore, the phase II CAESURA trial (NCT03912402) assessed the efficacy and safety of prolgolimab combined with platinum-based chemotherapy and bevacizumab in advanced cervical cancer (Fogt et al., 2023). The objective response rate was 63.8% per RECIST 1.1 criteria, with two complete and 35 partial responses. Under iRECIST criteria, the response rate improved to 70.7%, including the same two complete and an increased 39 partial responses. At 12 months, progression-free survival was recorded at 8.5 months per RECIST 1.1 and 13.1 months per iRECIST. The median overall survival had not yet been reached. Notably, 98% of participants experienced adverse events, with 69% considered treatment-related. There were 12 severe events (grade 3 or higher), and immune-related adverse events were noted in approximately 38% of patients. Despite these challenges, the trial demonstrated that the combination of prolgolimab, chemotherapy, and bevacizumab offers promising efficacy and an acceptable safety profile for treating advanced cervical cancer. A phase III placebo-controlled trial (NCT03912402) exploring this treatment regimen as a potential first-line therapy is currently underway, with results highly anticipated by the oncological community.

While the KEYNOTE and CAESURA studies underscore the effectiveness of PD-1 inhibitors in managing A/R/M cervical cancer, locally advanced cervical cancer (LACC) remains a significant clinical challenge. Despite innovative treatments, the relapse rate for LACC exceeds 40% following standard chemoradiation. This underscores the urgent need for improved therapeutic strategies in this setting. Notably, an elevated CD8+/FOXP3+ ratio in tumor cells post-neoadjuvant chemotherapy has been associated with better clinical outcomes (Liang et al., 2018), indicating potential biomarkers for therapeutic efficacy. The use of immune checkpoint inhibitors (ICIs) has shown survival benefits in recurrent cases (Colombo et al., 2021; Tewari et al., 2022). However, the CALLA trial did not demonstrate a significant prolongation in progression-free survival for high-risk LACC patients treated with adjuvant defactinib compared to radiotherapy and chemotherapy alone. This highlights the variability in response to new treatments and the need for further investigation. The COLIBRI trial (NCT04256213), as reported by France’s Isabelle Ray-Coquard (Ray-Coquard et al., 2023), focuses on the pre-radio-chemotherapy effects of dual immune therapy with nivolumab and ipilimumab in patients with cervical squamous cell carcinoma stages FIGO IB3-IVA. This trial confirmed the safety and potential efficacy of using nivolumab and ipilimumab as preemptive neoadjuvant treatments, followed by sustained nivolumab monotherapy after radio-chemotherapy. Significant increases in CD8^+^ cell proliferation, the CD8+/FOXP3+ ratio, and Histo-Cytometry Optimization Threshold (HOT) scores were observed after this dual immune therapy, analyzed using multi-lF or HTG technologies. These findings advocate for the continued exploration of ICIs as a neoadjuvant and sequential treatment strategy in LACC management. The initial results from the COLIBRI trial provide a promising basis for future integrated ICI strategies, suggesting a potential paradigm shift that could enhance survival rates and improve treatment outcomes for LACC patients. This ongoing research highlights the dynamic nature of cancer treatment innovations and the need for tailored strategies to address different stages and types of cervical cancer.

The inclusion of an anti-CTLA-4 antibody alongside PD-1 inhibition has notably extended the duration of response (DoR) and survival, resulting in an increased overall response rate (ORR) (Larkin et al., 2019; Xu et al., 2022). However, this combination therapy significantly raises the frequency of immune-related adverse events (irAEs) compared to anti-PD-1 monotherapy (Hellmann et al., 2019). Recently, alternative strategies have been explored, including a bispecific antibody capable of binding two different antigens or the same antigen at different epitopes, showing substantial promise in clinical trials (Wu et al., 2021; Wang et al., 2022; Wu et al., 2022). Another innovative approach involves QL1706 (PSB205), a bifunctional PD-1/CTLA-4 dual blocker. This engineered monoclonal antibody combines anti-CTLA-4 IgG1 with a novel bifunctional MabPair platform. QL1706 is characterized by a shortened elimination half-life (t1/2) for the CTLA-4 component, which may enhance tolerability by maintaining prolonged anti-PD-1 activity while reducing exposure to CTLA-4, potentially allowing patients to continue treatment longer without severe CTLA-4-related adverse effects. In a phase I trial on advanced solid tumors, including pretreated advanced cervical cancer, QL1706 demonstrated an ORR of 27.3% (15/55) with a median duration of response (mDoR) not reached; these results are comparable to the ORR of 25.6% reported for the dual PD-1 and CTLA-4 checkpoint blockade with balstilimab and zalifrelimab (O'Malley et al., 2022). As the first of its kind, this bifunctional MabPair product has shown promising antitumor activity and a favorable safety profile, indicating the potential for further development as a foundational agent in dual immunotherapy. This opens the door to the integration of multiple immunotherapeutic strategies, which could significantly enhance effectiveness. Such an approach may involve amplifying antigen-specific T cells, mitigating immunosuppression in the tumor microenvironment, or enhancing effector immune functions, potentially in combination with traditional treatments like chemotherapy or radiotherapy. This holistic immunotherapy approach could prove more effective than focusing on a single strategy, offering a robust framework for combating cancer.

Immunotherapy, particularly targeting the PD-1/PD-L1 pathway, has markedly transformed the treatment landscape for solid tumors, including A/R/M cervical cancers (Motzer et al., 2019; Herbst et al., 2020; An et al., 2022). Frequently, targeting a single signaling pathway does not sufficiently restore robust antitumor immunity, leading to resistance and recurrence (Yi et al., 2018). This is partly due to various factors within the tumor microenvironment (TME) that suppress immune responses, among which transforming growth factor-beta (TGF-β) plays a critical role (Batlle et al., 2019). TGF-β functions as a tumor suppressor in early-stage breast cancers by inducing cell cycle arrest and apoptosis (Moses et al., 2011). However, in advanced cancers, it paradoxically promotes tumor growth by facilitating metastasis, resistance to radiotherapy and chemotherapy, and remodeling the TME (Colak et al., 2017; Yi et al., 2022). TGF-β also extensively suppresses immune activities in the TME, affecting tumor-infiltrating lymphocytes (TILs), macrophage polarization, regulatory T cell (Treg) differentiation, and dendritic cell (DC) activity (Bagati et al., 2021). Additionally, it enhances the production of peritumoral collagen by cancer-associated fibroblasts (CAFs), which hinders the movement of TILs (Trimble et al., 2015). To overcome immunotherapy resistance, strategies that inhibit both PD-1 and TGF-β pathways, such as the use of bifunctional antibodies like M7824 and SHR1701, are gaining attention (Khalili-Tanha et al., 2023; Tan et al., 2022). These second-generation bifunctional agents, targeting both the TGF-β and PD-1 pathways, have been developed as fusion proteins targeting PD-L1/TGF-β. Preclinical studies suggest that M7824, for instance, not only suppresses tumor growth but also significantly enhances both innate and adaptive immunity more effectively than PD-1 inhibitors alone (Lan et al., 2018). Initial clinical trials highlighted M7824s potent anticancer capabilities (Yi et al., 2022). However, subsequent phase 2/3 trials in specific cancers such as bile duct and non-small-cell lung cancers have shown less efficacy than anticipated (Lind et al., 2020). The reasons for these discrepancies are not fully understood, but enhancing the identification of responsive patients through predictive markers could significantly improve the effectiveness of these bifunctional antibodies. This approach promises to refine and optimize the therapeutic strategies in immunotherapy, potentially leading to more personalized and effective cancer treatments.

The immune normalization approach focuses on restoring impaired anti-tumor immune responses by targeting key pathways such as PD-1/PD-L1 to modify the TME (Sanmamed et al., 2018; Bai, et al., 2019). However, for most patients, the immune imbalance in the TME is complex, and addressing additional abnormalities is often crucial to overcome resistance to PD-1/PD-L1 inhibitors. In this context, the development of bispecific antibodies, such as YM101, which targets both PD-L1 and murine TGF-β with a ScFv-IgG-like structure, represents a significant advancement (Yi et al., 2021). YM101 is designed to mitigate the dominant inhibitory effects of TGF-β, shifting the “cancer-immunity set point” from immune tolerance to active T cell immunity, enhancing the therapeutic landscape. Functional assays have demonstrated that YM101 effectively counters the suppressive actions of both the PD-1 and TGF-β pathways. *In vivo* studies reveal that YM101 significantly reduces tumor growth in mouse models, affirming its potential as a dual-targeting therapeutic agent. Moreover, YM101 has been shown to be both effective and safe in preclinical evaluations (Yi et al., 2021). Another analogous bispecific molecule, BiTP, tailored for further clinical investigations, exhibits high affinity for its targets and effectively inhibits subsequent signaling pathways (Yi et al., 2022). Preclinical tests indicate that BiTP can slow tumor growth and extend survival in animal models, showing particular promise against triple-negative breast cancer (Yi et al., 2022). In the TME, BiTP is noted for reducing stromal collagen buildup, enhancing T cell penetration, and diminishing immune-suppressive elements, suggesting a robust candidate for advancing to clinical trials. Additionally, TQB2858, another bispecific antibody targeting PD-L1 and TGF-β, is currently under clinical evaluation and demonstrates potential as a therapeutic option (Xing et al., 2023). The evolution of anti-PD-L1/TGF-β bispecific antibodies signifies a major shift from traditional PD-1/PD-L1 monoclonal antibodies, offering a synergistic approach that may transform immune-excluded tumors into immune-inflamed ones, thereby amplifying the efficacy of existing therapies and broadening the horizons of immunotherapy. While initial results are promising, extensive studies are required to fully ascertain the therapeutic potential of TGF-β targeting in cervical cancer treatment. Furthermore, the integration of TGF-β inhibitors with other immunotherapeutic strategies, such as vaccines or cell-based treatments, could potentially enhance overall therapeutic outcomes, providing a comprehensive approach to combating this malignancy.

### 4.4 Limitations

This study represents the first bibliometric analysis of original articles focused on immunotherapy for A/R/M cervical cancer. By conducting a quantitative review of the literature, this research summarizes the development of immunotherapy in this field and aims to guide future scholarly inquiries. However, there are several limitations to consider. Firstly, the exclusive use of the Web of Science database for data collection may introduce a publication bias. Moreover, the restriction to English-language publications could have introduced a language-specific publication bias, potentially overlooking relevant studies published in other languages. Additionally, using bibliometric tools that are based on artificial intelligence and computational linguistics, as suggested by earlier research (Yan et al., 2021), could make it harder to get all the information that is needed. Even with these problems, our results are mostly the same as those of recent traditional reviews (Roden and Stern, 2018; Ferrall et al., 2021). These results give researchers more unbiased information that helps them understand and from see things a different point of view in the field of immunotherapy for A/R/M cervical cancer. These limitations underscore the need for broader and more inclusive research methodologies that expand beyond single-database searches and English-language constraints to ensure a more comprehensive global perspective in future bibliometric analyses.

## 5 Conclusion

In general, this work employs a methodical bibliometric approach to analyze the articles on immunotherapy for advanced, recurrent, and metastatic cervical cancer. It offers useful insights through quantitative evaluations and knowledge mapping. In order to advance research, it will be crucial to strengthen international and institutional relationships in the future. Currently, there is a strong focus on enhancing our knowledge of the immune features of A/R/M cervical cancer, identifying patient populations that are most likely to respond well to immunotherapy, and exploring the practical uses of therapeutic vaccines, adoptive T cell therapies (ACT), immune checkpoint inhibitors (ICIs), and innovative dual-specificity treatments that target both TGF-beta and PD-L1. These therapeutic techniques are progressively being integrated with conventional treatments such as chemotherapy, radiation, and targeted therapies. The future of cervical cancer immunotherapy will likely include enhancing treatment effectiveness, minimizing adverse effects, and developing more powerful, individualized treatment strategies that cater to different disease phases.

## Data Availability

The raw data supporting the conclusion of this article will be made available by the authors, without undue reservation.
