# Supplementary material for: A comprehensive bibliometric analysis (2000–2022) on the mapping of knowledge regarding immunotherapeutic treatments for advanced, recurrent, or metastatic cervical cancer
Source: Front Pharmacol. 2024 May 10;15:1351363. doi: 10.3389/fphar.2024.1351363 (PMC11116801; doi:10.3389/fphar.2024.1351363)
Supplement: Supplementary file 1 [file Table1.DOCX]

**Supplementary Table 1 The top 10 journals of immunotherapy for A/R/M cervical cancer**

| **Rank** | **Journal** | **N (%)** | **Citations** | **IF (2022)** | **H-Index**  **(2021)** | **JCR**  **division** | **Country** |
| --- | --- | --- | --- | --- | --- | --- | --- |
| **1** | International Journal of Cancer | 31(3.1%) | 1355 | 6.4 | 212 | Q1 | Switzerland |
| **2** | Cancer Immunology Immunotherapy | 29(2.9%) | 1068 | 5.8 | 104 | Q3 | USA |
| **3** | Vaccine | 28(2.8%) | 1536 | 5.5 | 164 | Q3 | England |
| **4** | Gynecologic Oncology | 26(2.6%) | 637 | 4.7 | 147 | Q1 | USA |
| **5** | Oncoimmunology | 21(2.1%) | 722 | 7.2 | 58 | Q1 | USA |
| **6** | Frontiers in Immunology | 20(2.0%) | 299 | 7.3 | 84 | Q1 | Switzerland |
| **7** | Frontiers in Oncology | 20(2.0%) | 123 | 4.7 | 60 | Q2 | Switzerland |
| **8** | Cancer Research | 19(1.9%) | 1357 | 11.2 | 411 | Q1 | USA |
| **9** | Clinical Cancer Research | 17(1.7%) | 1508 | 11.5 | 292 | Q1 | USA |
| **10** | International Journal of Gynecological Cancer | 17(1.7%) | 433 | 4.8 | 79 | Q2 | USA |

N: number of publications; % of total publications.
