# Supplementary material for: A comprehensive bibliometric analysis (2000–2022) on the mapping of knowledge regarding immunotherapeutic treatments for advanced, recurrent, or metastatic cervical cancer
Source: Front Pharmacol. 2024 May 10;15:1351363. doi: 10.3389/fphar.2024.1351363 (PMC11116801; doi:10.3389/fphar.2024.1351363)
Supplement: Supplementary file 2 [file Table2.DOCX]

**Supplementary Table 2 The top 10 cited journals of immunotherapy for A/R/M cervical cancer**

| **Rank** | **Journal** | **Cited Number** | **IF (2022)** | **H-Index**  **(2021)** | **JCR**  **division** | **Country** |
| --- | --- | --- | --- | --- | --- | --- |
| **1** | Cancer Research | 630 | 11.2 | 411 | Q1 | USA |
| **2** | Clinical Cancer Research | 539 | 11.5 | 292 | Q1 | USA |
| **3** | International Journal of Cancer | 496 | 6.4 | 212 | Q1 | Switzerland |
| **4** | Journal of Immunology | 479 | 4.4 | 345 | Q2 | USA |
| **5** | Proceedings of the National Academy of Sciences of the United States of America | 440 | 11.1 | 699 | Q1 | USA |
| **6** | New England journal of Medicine | 413 | 158.5 | 933 | Q1 | USA |
| **7** | Nature | 373 | 64.8 | 1096 | Q1 | England |
| **8** | Journal of Clinical Oncology | 356 | 45.3 | 494 | Q1 | USA |
| **9** | Science | 338 | 56.9 | 1058 | Q1 | USA |
| **10** | Cancer Immunology Immunotherapy | 325 | 5.8 | 104 | Q2 | USA |
