# Supplementary material for: A comprehensive bibliometric analysis (2000–2022) on the mapping of knowledge regarding immunotherapeutic treatments for advanced, recurrent, or metastatic cervical cancer
Source: Front Pharmacol. 2024 May 10;15:1351363. doi: 10.3389/fphar.2024.1351363 (PMC11116801; doi:10.3389/fphar.2024.1351363)
Supplement: Supplementary file 3 [file Table3.DOCX]

**Supplementary Table 3 The top 10 most productive authors of immunotherapy for A/R/M cervical cancer**

| **Rank** | **Author** | **Article count** | **Total number of citations** | **Total link strength** | **Average number of citations** | **H-Index**  **(2021)** |
| --- | --- | --- | --- | --- | --- | --- |
| **1** | Van der Burg SH | 25 | 1878 | 1365 | 75.12 | 81 |
| **2** | Kenter GG | 16 | 1716 | 985 | 107.25 | 56 |
| **3** | Welters MJP | 14 | 981 | 928 | 70.07 | 43 |
| **4** | Melief CJM | 12 | 1376 | 863 | 114.67 | 103 |
| **5** | Hung CF | 10 | 423 | 246 | 42.30 | 67 |
| **6** | Jordanova ES | 10 | 878 | 324 | 87.80 | 47 |
| **7** | Nijman HW | 8 | 418 | 264 | 52.25 | 49 |
| **8** | Fleuren GJ | 8 | 1078 | 674 | 134.75 | 68 |
| **9** | Oostendorp J | 8 | 784 | 511 | 98 | 20 |
| **10** | Hinrichs CS | 8 | 838 | 263 | 104.75 | 31 |
