# Supplementary material for: A comprehensive bibliometric analysis (2000–2022) on the mapping of knowledge regarding immunotherapeutic treatments for advanced, recurrent, or metastatic cervical cancer
Source: Front Pharmacol. 2024 May 10;15:1351363. doi: 10.3389/fphar.2024.1351363 (PMC11116801; doi:10.3389/fphar.2024.1351363)
Supplement: Supplementary file 4 [file Table4.DOCX]

**Supplementary Table 4 The top 10 co-cited references related to immunotherapy for A/R/M cervical cancer**

| **Rank** | **Citation**  **Counts** | **The title of Article** | **Year** | **Journal** | | | |
| --- | --- | --- | --- | --- | --- | --- | --- |
|  |  |  |  | **Name** | **Country** | **IF**  **(2022)** | **H-Index**  **(2021)** |
| **1** | 70 | Global cancer statistics 2018: GLOBOCAN estimates of incidence and mortality worldwide for 36 cancers in 185 countries | 2018 | CA-A Cancer Journal for Clinicians | USA | 254.7 | 144 |
| **2** | 64 | Efficacy and Safety of Pembrolizumab in Previously Treated Advanced Cervical Cancer: Results from the Phase II KEYNOTE-158 Study | 2019 | Journal of Clinical Oncology | USA | 45.3 | 494 |
| **3** | 53 | Safety and Efficacy of Pembrolizumab in Advanced, Programmed Death Ligand 1-Positive Cervical Cancer: Results from the Phase Ib KEYNOTE-028 Trial | 2017 | Journal of Clinical Oncology | USA | 45.3 | 494 |
| **4** | 31 | Safety and Efficacy of Nivolumab Monotherapy in Recurrent or Metastatic Cervical, Vaginal, or Vulvar Carcinoma: Results from the Phase I/II Checkmate 358 Trial | 2019 | Journal of Clinical Oncology | USA | 45.3 | 494 |
| **5** | 31 | Complete regression of metastatic cervical cancer after treatment with human papillomavirus-targeted tumor-infiltrating T cells | 2015 | Journal of Clinical Oncology | USA | 45.3 | 494 |
| **6** | 30 | Cervical Cancer | 2019 | Lancet | USA | 168.9 | 700 |
| **7** | 30 | Integrated genomic and molecular characterization of cervical cancer | 2017 | Nature | England | 64.8 | 1096 |
| **8** | 27 | Vaccination against HPV-16 oncoproteins for vulvar intraepithelial neoplasia | 2009 | The New England Journal of Medicine | USA | 158.5 | 933 |
| **9** | 25 | Induction of tumour-specific CD4+ and CD8+ T-cell immunity in cervical cancer patients by a human papillomavirus type 16 E6 and E7 long peptides vaccine | 2008 | Clinical Cancer Research | USA | 11.5 | 292 |
| **10** | 21 | Prognostic effect of different PD-L1 expression patterns in squamous cell carcinoma and adenocarcinoma of the cervix | 2016 | Modern Pathology | USA | 7.5 | 139 |
